# Ossified Gall Bladder

**Published:** 1848-03

**Authors:** 


*Ossified Gall-Bladder.*—Mr. Bagshaw exhibited to the Bath Pathological Society an ossified gall-bladder. The individual from whom the specimen was taken, was a woman aged 70. There had never been any particular symptoms referable to the liver, except occasional bilious vomiting; the secretion of bile had always been abundant, and the appetite for food at times craving. The coats of the gall-bladder were from an eighth to a quarter of an inch in thickness, and contained throughout an abundant deposit of calcareous matter, except at one or two points, where the deposit had not taken place. The gall-bladder contained a quantity of yellowish-white, pasty looking matter, which yielded about 40 per cent. of cholesterine. Mr. Bagshaw remarked on the case, as being one of considerable rarity.—*Ibid.*

